# Non-Celiac Gluten Sensitivity Has Narrowed the Spectrum of Irritable Bowel Syndrome: A Double-Blind Randomized Placebo-Controlled Trial

**DOI:** 10.3390/nu7064542

**Published:** 2015-06-05

**Authors:** Bijan Shahbazkhani, Amirsaeid Sadeghi, Reza Malekzadeh, Fatima Khatavi, Mehrnoosh Etemadi, Ebrahim Kalantri, Mohammad Rostami-Nejad, Kamran Rostami

**Affiliations:** 1 Gastroenterology Unit, Imam Khomeini Hospital, Tehran University of Medical Sciences, Tehran 5715915199, Iran; E-Mail: bijan.shahbaz@gmail.com; 2 Digestive Disease Research Center, Digestive Disease Research Institute, Tehran University of Medical Sciences, Shariati Hospital, Tehran 1599666615, Iran; E-Mail: dr.reza.malekzadeh@gmail.com; 3 Sasan Alborz Biomedical Research Center, Masoud Gastroenterology and Hepatology Clinic, Tehran 14117-13135, Iran; 4 Students’ Scientific Research Center, Tehran University of Medical Sciences, Tehran 1449614535, Iran; E-Mails: hototo24@yahoo.com (F.K.); Mehr_etemadi@yahoo.com (M.E.); 5 Gholhak Medical Laboratory, Tehran 1913913948, Iran; E-Mail: Kalantri@yahoo.com; 6 Gastroenterology and Liver Diseases Research Center, Research Institute for Gastroenterology and Liver Diseases, Shahid Beheshti University of Medical Sciences, Tehran 1985714711, Iran; E-Mail: m.rostamii@gmail.com; 7 Department of Gastroenterology, Alexandra Hospital, Worcestershire B98 7UB, UK; E-Mail: kamran.rostami@nhs.net

**Keywords:** IBS, non-celiac gluten sensitivity, gluten free diet

## Abstract

Several studies have shown that a large number of patients who are fulfilling the criteria for irritable bowel syndrome (IBS) are sensitive to gluten. The aim of this study was to evaluate the effect of a gluten-free diet on gastrointestinal symptoms in patients with IBS. In this double-blind randomized, placebo-controlled trial, 148 IBS patients fulfilling the Rome III criteria were enrolled between 2011 and 2013. However, only 72 out of the 148 commenced on a gluten-free diet for up to six weeks and completed the study; clinical symptoms were recorded biweekly using a standard visual analogue scale (VAS). In the second stage after six weeks, patients whose symptoms improved to an acceptable level were randomly divided into two groups; patients either received packages containing powdered gluten (35 cases) or patients received placebo (gluten free powder) (37 cases). Overall, the symptomatic improvement was statistically different in the gluten-containing group compared with placebo group in 9 (25.7%), and 31 (83.8%) patients respectively (*p* < 0.001). A large number of patients labelled as irritable bowel syndrome are sensitive to gluten. Using the term of IBS can therefore be misleading and may deviate and postpone the application of an effective and well-targeted treatment strategy in gluten sensitive patients.

## 1. Introduction

Dietary therapies are gaining popularity as evidence of efficacy for specific diets have emerged. The symptoms attributed to irritable bowel syndrome (IBS) seem to have a different etiology, and it has been reported that many patients fulfilling the criteria for IBS have, in fact, a kind of sensitivity to some nutrient components like FODMAP (Fermentable Oligo-Di-Monosaccharides and Polyols), gluten or lactose. Other major groups develop their symptoms related to anxiety, depression and work-related stresses. It is very clear that the spectrum of IBS is narrowing by the recent advances in diagnostic tools [1]. Many conditions that were called IBS 20 years ago, do have a clear and treatable etiology. This is why IBS has a changeable definition in different settings [2]. The major overlooked etiologies that are labelled as irritable bowel syndrome are food sensitivities, anxiety and depression. Due to a lack of proper diagnosis, often depression becomes a part of the natural history in sufferers with symptoms attributed to IBS [3,4,5,6]. There are at least three postulated mechanisms by which food components might induce functional gut symptoms in IBS. These three mechanisms are: immune mediated/mast cell pathway (usually referred as “food hypersensitivity”), direct action of bioactive molecules (generally referred as to “food chemicals”) and luminal distension [4].

Severe gluten sensitivity without any damage to the intestinal mucosa was reported in some patients fulfilling the criteria for IBS [5]. It is still not very clear how gluten sensitivity causes a range of symptoms in these patients [7]. Some patients who are labelled as post gastroenteritis IBS may in fact have food sensitivity [4].

Recent studies have found that patients with so-called IBS suffer from non-celiac gluten sensitivity and their IBS symptoms improved after six weeks of a gluten-free diet [6,7,8]. Medical practices only recently started to focus attention on the pathogenicity of some nutrients in causing gastrointestinal symptoms, and it is disappointing that health care professionals receive little formal training in the dietary management of IBS and have traditionally viewed dietary interventions with skepticism. The food sensitivity behind the symptoms caused by changes in motility, visceral sensation, microbiome, permeability, immune activation, and brain-gut interactions are key elements in the pathogenesis of food sensitivity previously attributed to IBS. The role of specific dietary modification in the management of this group of patients has not been rigorously investigated until recently. There is now credible evidence suggesting that targeted dietary carbohydrate exclusion provides clinical benefits in unrecognised food-sensitive patients masked under the diagnosis of IBS. There is emerging evidence to suggest that proteins such as gluten, as well as food chemicals, may play a cardinal role in the symptoms of these patients [9].

We hope, by undertaking the step to evaluate the role of gluten-free diet in patients treated symptomatically under the diagnosis of IBS, to prevent the side effects of medications used for symptomatic relief in those patients who, in fact, are gluten-sensitive.

The aim of this study was thus to evaluate the effects of a gluten-free diet in Iranian patients with a diagnosis of IBS to assess whether dietary intervention has any role in the treatment package of these patients.

## 2. Materials and Methods

### 2.1. Study Population

During the years 2011–2013, 148 patients with newly diagnosed IBS based on the Rome III criteria [10] and more than 16 years of age, were recruited in this double-blind randomized placebo-controlled trial study (DBRP). All participants were recruited from a suburban, outpatient, private-practice gastroenterology clinic in Imam Khomeini hospital, Tehran, Iran.

### 2.2. Inclusion and Exclusion Criteria

From this number 46 patients were excluded from the study because of the following criteria and associations: subjects who had a known diagnosis of celiac disease (CD) and had ever tried a gluten free diet (GFD) and whether this diet was currently in place; patients with self-exclusion of wheat from the diet without a known diagnosis of CD; patients with inflammatory bowel disease and diabetes; those who used any concurrent drugs for depression and/or anxiety; people who used non-steroidal anti-inflammatory drugs; subjects with abnormal levels of: glucose, urea, creatinine, sodium, potassium, hemoglobin, ESR (erythrocyte sedimentation rate) and thyroid function tests; and those who did not sign the consent form to participate in the study.

CD was excluded as a diagnosis in all patients through a combination of serological testing (anti-Tissue transglutaminase antibody (anti-tTG Ab), total IgA levels, and/or anti-endomysial antibody (anti-EMA Ab) positive test) (three cases), and villi atrophy at the duodenal histology (1 case) performed at endoscopy while on a gluten-containing diet. Also those with positive IgE-mediated immuno-allergy tests to wheat were excluded. Only those patients who fulfilled the criteria recently proposed by the experts’ meeting on “gluten sensitivity” [11,12] were included in the present study. All were negative for CD serology (anti-endomysial antibody and anti-Tissue transglutaminase antibody of IgA class), wheat allergy tests (specific IgE and skin prick tests) and normal duodenal biopsy with preserved villous architecture performed at the time of endoscopy on a gluten-containing diet.

### 2.3. Clinical Trial

The gluten free diet was started in 102 IBS patients; 22 patients found it hard and difficult to continue with the gluten-free diet and subsequently were withdrawn from study. Eighty patients responded to the diet and achieved significant improvement. From this group 8/80 did not follow a strict gluten free diet and were unwilling to continue the diet any further, and the remaining 72 patients completed the study. At the end of phase one, 72 patients met the inclusion criteria. From this group, 35 out of 72 were randomized in the gluten group and 37 out of 72 were in the placebo group for six weeks. The mean age in the gluten group was 44.5 ± 10 years and 43.2 ± 17 years in the placebo group. Six patients (17.1%) in the gluten group and 13 in the placebo group (35.1%) were male.

Patients were asked to complete a symptom questionnaire containing the question for the primary outcomes including bloating, abdominal pain, defecation satisfaction, nausea, fatigue and overall symptoms, and scored with visual analogue scale (VAS), with 0 representing no symptoms and 10 indicating severe clinical signs and symptoms.

The patients were randomized according to block randomization method held by an independent observer to either the gluten or the placebo treatment group. Both patients and investigators evaluating patients were blinded to the study treatment. After randomization, serum markers were measured for antibodies to tissue transglutaminase (*i.e.*, tissue transglutaminase IgA) and whole gliadin (IgA and IgG) by ELISA (enzyme-linked immunosorbent assay) using commercially available kits (AESKULISA tTG/AGA, Wendelsheim, Germany). According to the manufacturer’s reference ranges, serological results were considered negative when <10 IU/mL; higher values were considered positive.

### 2.4. Assessment of Dietary Compliance

**Figure 1:**
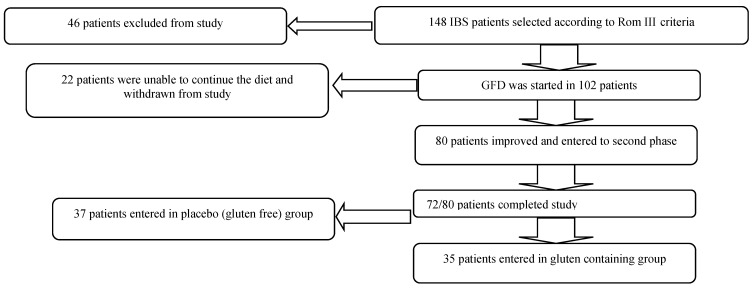
Recruitment pathway and reasons for screen failure and withdrawals.

At each weekly follow-up assessment, dietary adherence was evaluated by a dietitian assessing consumption of any gluten-containing nutrient. Dietary compliance was considered optimal if the consumption of gluten was below 100 mg/day. Those patients (8/80) who did not comply with this policy despite their improvement did not continue with study. After six weeks (phase two) 72/80 patients who complied with the diet optimally and made a significant improvement, agreed to continue with the study and be enrolled in double-blind randomized placebo-controlled trial challenges (Figure 1).

Group A, including 35 patients (the gluten containing group), was given a packet (100 g) containing a gluten meal (free of fermentable oligo di-monosaccharides and polyols and proteins including 2.3% non-gluten, 52% gluten and/or gliadin and 27.7 g glucose). Group B, including 37 patients (placebo group (gluten-free powder)), was given packets (100 g) containing powder of gluten-free foods (rice flour, corn starch and glucose). HLA typing was performed for all patients in both groups.

Patients in both groups consumed powder for six weeks, while both groups were on gluten-free diets. Packages each contained two packs of 50 g powder, each to be poured in a cup containing 150 mL warm water, stirring the emulsion immediately and consuming one with breakfast and one with dinner. Initially, the participants were given complete information about the process of study. The study was approved by the institutional ethics committees of the Gastroenterology Department, Imam Khomeini Hospital, Tehran University of Medical Sciences, Tehran (study number CT711; approval date: 22 November 2012), and all participants signed a written informed consent regarding participation in the research project.

### 2.5. Statistical Analysis

The results and VAS mean scores were analyzed by χ^2^ and Mann-Whitney U test, respectively using SPSS software version 15. Progressions of symptoms in both groups were examined by repeated measurement test. In this study predictive values less than 0.05 was recognized as significant.

## 3. Results

Seventy-two patients were evaluated into this double-blind randomized placebo-controlled trial. IBS type in the placebo group consisted of constipation type in six patients (16.2%), diarrhea type in 18 (48.6%) and mixed type in 13 (35.1%) patients. On the other hand, type of IBS in the gluten containing group consisted of constipation type in 10 (28.6%), diarrhea type in 19 (54.3%) and mixed type in 6 (17.1%) patients. No statistical differences were detected when the type of IBS in two groups (*p* = 0.089) were compared. The placebo group (62.1%) and gluten-containing group (48.5%) carried either DQ2 or DQ8 haplotypes. Table 1 summarizes the demographic and clinical characteristics of the gluten compared to placebo group. As the result shows there were no statistically significant differences between the groups regarding gender and mean age (*p* > 0.05).

The statistical analysis showed that the differences between gluten-containing and placebo groups in the overall symptoms (placebo (16.2%), gluten (74.3%)) including satisfaction with stool consistency (placebo (8.1%), gluten (77.1%)), tiredness (placebo (8.8%), gluten (60%)), nausea (placebo (5.4%), gluten (8.3%)), bloating (placebo (16.2%), gluten (74.3%)) were statistically significant (*p* = 0.001).

**Figure 2:**
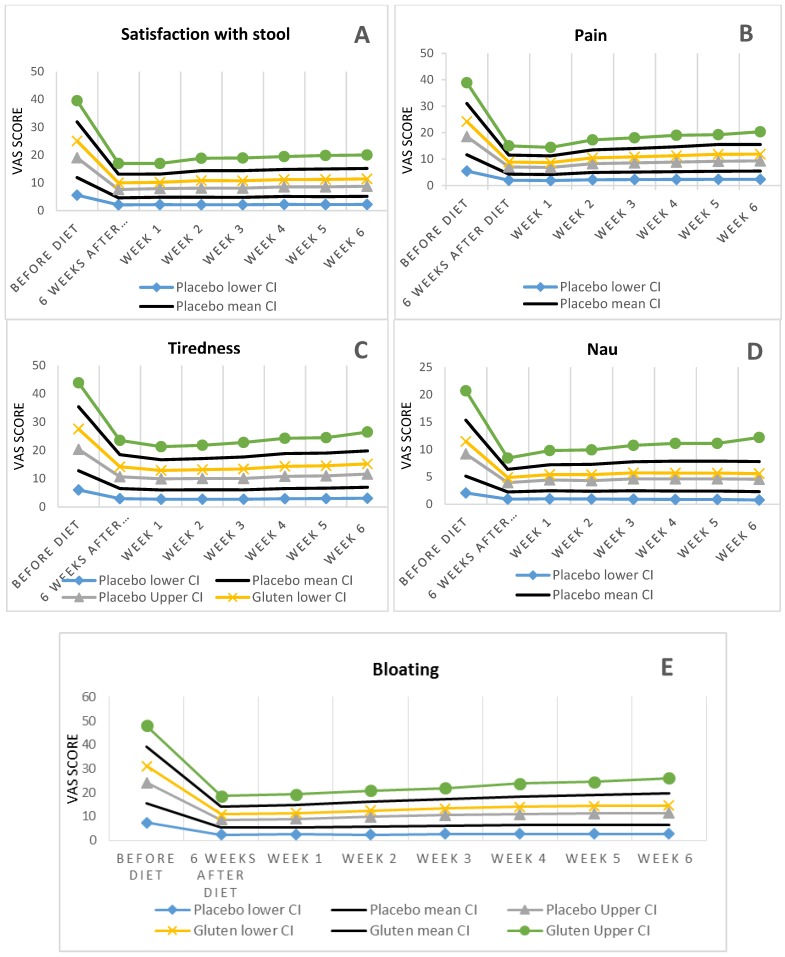
The difference between gluten and placebo groups in the pattern of symptoms. (**A**) Satisfaction with stool consistency; (**B**) Pain; (**C**) Tiredness; (**D**) Nausea; (**E**) Bloating.

After checking normality and Sphericity assumptions, repeated measurement test was employed to analyse the scores of symptoms, and revealed no statistically significant difference for symptom’s score including satisfaction with stool consistency (*p* = 0.15), tiredness (*p* = 0.6), nausea (*p* = 0.6), and bloating (*p* = 0.3) between placebo and gluten groups (See Figure 2).

After six weeks of the diet, symptoms were controlled only in nine patients (25.7%) in the gluten-containing group, compared to 31 patients (83.8%) in the placebo group, indicating that 26 out of 35 patients in the gluten group became symptomatic on gluten-challenge. Comparison of these ratios indicated that there were significant differences between gluten-containing and placebo group symptoms control (*p* < 0.001). In the gluten-containing group, all symptoms significantly increased especially for bloating and abdominal pain (from 3.1 ± 2.3 to 5.1 ± 2.2) one week after starting the gluten. Mean bloating VAS before starting the gluten-free diet in the gluten group was 8.4 ± 1.5, and after the six-week diet was decreased to 3.1 ± 2.3. Overall time trend analysis revealed statistically significant satisfaction with stool consistency (*p* = 0.01) and bloating (*p* = 0.05) (but no statistically significant difference between the two groups). After a one-week gluten challenge, the mean score was increased to 4 ± 2.1, which was not statistically significant compared with the placebo group. But this symptom continued to increase to 5.1 ± 2.2 in the fifth week.

At the beginning of the study three patients in the placebo and none in the gluten group were positive for anti-gliadin antibodies (AGA) IgG. At the end of six weeks, the patients were tested for anti-gliadin antibodies (IgG and IgA) and tissue transglutaminase antibodies (IgG and IgA) (Table 1). Except for one patient, who had increased anti-gliadin antibodies IgG titer, the titers of the rest of the patients were unchanged, and therefore, no significant differences were seen before and after the regimen in either of the two groups (*p* > 0.05).

**Table 1:** Demographic and clinical characteristics of the gluten compared to placebo group.

Antibody	Placebo	95% CI ^*^	Gluten	95% CI	*p* Value
Number of patients	37		35		**--**
Age average	43.2 ± 17	37.6–8.8	44.5 ± 10	41.11–47.89	**0.241**
Frequency (%) of male gender	13 (35.1%)	19%–51%	6 (17.1%)	4%–30%	**0.083**
**Kind of IBS**					**0.089**
Constipation	6 (16.2%) [4%–28%]		10 (28.6%) [13%–43%]		
Diarrhea	18 (48.6%) [32.6%–64.6%]		19 (54.3%) [37%–71%]		
Mixed	13 (35.1%) [20%–50%]		6 (17.1%) [5%–29%]		
Tiredness	(8.8%)		(60%)		
Pain	(16.2%)		(76.3%)		
^1^ Anti-tTG IgA	2.3 ± 0.4	2.17–2.43	2.5 ± 0.4	2.37–2.63	**0.259**
Anti-tTG IgG	2 ± 0.3	1.02–2.98	2.2 ± 0.3	2.1–2.3	**0.174**
^2^ AGA IgA	2.9 ± 1.8	2.31–3.49	2.6 ± 0.5	2.43–2.77	**0.110**
AGA IgG	2.6 ± 1.4	2.14–3.06	2.4 ± 0.4	2.27–2.53	**0.119**
Positive DQ2/8 ^3^ HLA	23 (62.1%)	46%–78%	17 (48.5%)	31%–65%	**0.792**

^1^ Anti-tTG: anti- tissue transglutaminase antibodies; ^2^ AGA: anti gliadin antibodies; ^3^ HLA: human leukocyte antigen; CI: In statistics, a confidence interval (CI) is a type of interval estimate of a population parameter.

## 4. Discussion

The relationship between nutrition and the gut environment is complex and has been a long-standing subject of intense debate. Although the effects of food allergies such as wheat consumption on gut motility have been well studied, the role of non-allergic, immune hypersensitivity reactions to specific food components is less understood [13,14,15]. In this study we have clarified that many cases treated symptomatically under the IBS label do have gluten sensitivity. Distinguishing between a non-specific terminology like IBS, and gluten sensitivity is extremely important since the later could be treated specifically and effectively with gluten restriction without implementing a long-term symptomatic drug therapy and having the consequences of drug side effects.

Gluten intolerance in people without celiac disease is a common condition, and most recently was described as “non-celiac gluten sensitivity” [12]. Gastrointestinal signs and symptoms in many patients labelled as IBS seem to be improved after exclusion of gluten from their diet. Basic evidence for such a claim is based on few randomized controlled trials [7,16,17,18].

This double-blind randomized placebo-controlled trial was performed on the patients with a diagnosis of IBS presenting with intestinal and extra-intestinal symptoms and confirmed that the differences between gluten-containing and placebo groups in the overall symptoms control was statistically significant.

It has become increasingly clear that intestinal inflammation may provide an initial stimulus for a persistent state of visceral hypersensitivity [19]. In fact, it might be that the microenteropathy [20] related to primary or acquired [4] e.g., post gastroenteritis food sensitivity, may cause intestinal irritability.

The role of inflammation in chronic abdominal pain comes from studies evaluating patients with gastroenteritis. Colonic biopsies of patients with persistent symptoms after a gastroenteritis reveal no signs of overt inflammation but show persistent minor increases in epithelial T lymphocytes and mast cells, [21] suggesting that long-term inflammatory changes may be responsible for colonic hypersensitivity.

Identifying and treating this state of hypersensitivity with dietary intervention has opened a new prospect in recognising food sensitivities and bringing IBS to an end as a non-specific and unuseful diagnosis. Unfortunately dietary intervention might not be convenient for every patient in particular in countries where the availability of gluten free products is limited. A significant number of our study group left the study as they were not able to cope with the dietary restrictions.

Abdominal pain was a common presentation in the remaining 72 patients and dietary intervention scored high in resolution of this symptom. Other GI symptoms including bloating and abdominal pain were raised in six patients out of 37 patients in the placebo group and in 26 out of 35 patients in the gluten group. Some studies suggest that pain might be related to wheat’s insoluble fibre that may causes increased gas, bloating and cramping after inducing an inflammatory reaction, activating the mast cells in proximity of colonic nerves, and releasing the mediators such as serotonin, histamine and proteases, which are able to activate visceral afferent nerve fibres [22,23]. A correlation might also exist between mast cell degranulation and IgE level in some cases. But the biological activity of some allergen types may also be affected by other parameters [24]. Similar to our finding, Biesiekierski *et al*. [7], reported the reappearance of extra-intestinal symptoms in the group that was re-challenged with gluten than those receiving placebo. It is well known that, besides the intestinal phenotype characterized by IBS-like symptoms, gluten-sensitive patients have plenty of extra-intestinal manifestations, the most frequent being those related to the peripheral and central nervous system, skin and joint/muscle. In contrast to the well-described acknowledgement of extra-intestinal symptoms in cases with gluten-related disorders, extra intestinal symptoms are usually ignored in IBS patients.

Increased “tiredness” by gluten suggests that in addition to the effect of gluten on the intestinal mucosa permeability, it may have a systemic effect in gluten-sensitive patients. Significant difference between gluten-containing and placebo groups regarding this symptom in this study (*p* < 0.002) and also demonstrated in the Biesiekierski study (*p* < 0.001) [7] confirm this hypothesis. The same research group also confirmed in their recent study that the investigated patients remarkably improved on the GFD and their symptoms were well controlled [25].

Despite the difficulties and challenges related to following a gluten-free diet, close communication with participant and support provide by study team facilitated the plan to full compliance with the gluten-free diet. The patients were guided by facilitating access to reliable reference for gluten-free products.

In this study, we did rely only on clinical criteria to evaluate response to regimen, because food sensitivity diagnosis is based on clinical diagnostic criteria and investigation findings are important mainly for ruling out the other intestinal disorders.

There is a need for highly sensitive and specific biomarkers to identify [26] mild inflammation or injury and damages of the intestinal mucosa and systemic inflammation; such biomarkers may help to determine the role and mechanism of gluten in inducing intestinal and extra-intestinal symptoms. A recent study by Valerii *et al*. demonstrates that wheat protein can induce an over-activation of the pro-inflammatory chemokine CXCL10 in peripheral blood mononuclear cell (PBMC) from non-celiac gluten sensitivity patients [27].

Rapid increase of intestinal and extra-intestinal symptoms can occur within hours, days or weeks after the exposure to gluten. Therefore developing symptoms using gluten challenge provides a cardinal criterion of strong reliability for the diagnosis of non-celiac gluten sensitivity.

In our study the antibody titers did not reach statistical differences before and at the end of the dietary intervention similar to others [7].

Identifying the NCGS correctly by using Salerno criteria, would help to differentiate NCGS from IBS [28]. The differentiation between these conditions is important as fermentable carbohydrates delivered to the colon have potential anti carcinogenic and anti-inflammatory actions [29]. Restriction of FODMAPs delivered to the colon might consequently have adverse effects on colonic health. A randomized parallel group study evaluated the effects on fecal microbiota of a dietitian-taught low FODMAP diet compared with those of a habitual diet indicated a reduction of the proportion and concentration of *Bifidobacteria* spp., providing the first evidence for potentially unfavourable effects of the low FODMAP diet [30]. Even though the carbohydrate restriction is much less in GFD compared to the low FODMAP, it has been demonstrated that levels of *Bifidobacteria* and *Lactobacilli* are reduced in CD patients on GFD [31,32,33]. While effect of GFD on microbiota needs further clarification in NCGS, low FODMAP in the long term seems to be associated with reducing total bacterial abundance in the feces [34], and quite rightly, Halmos *et al*. suggest that the low FODMAP diet should be recommended cautiously and avoided in asymptomatic cases. Therefore, distinguishing between gluten sensitivity and other carbohydrate sensitivities may need to be the starting point in managing patients fulfilling the Rome III criteria as reduced FODMAP delivery to colonic microbiota might have deleterious effects on the growth of bacteria, and thus potentially unfavorable health effects. Implementing low FODMAP perhaps should be restricted to those patients with additional carbohydrate sensitivity induced by FODMAP only.

## 5. Conclusions

**Figure 3:**
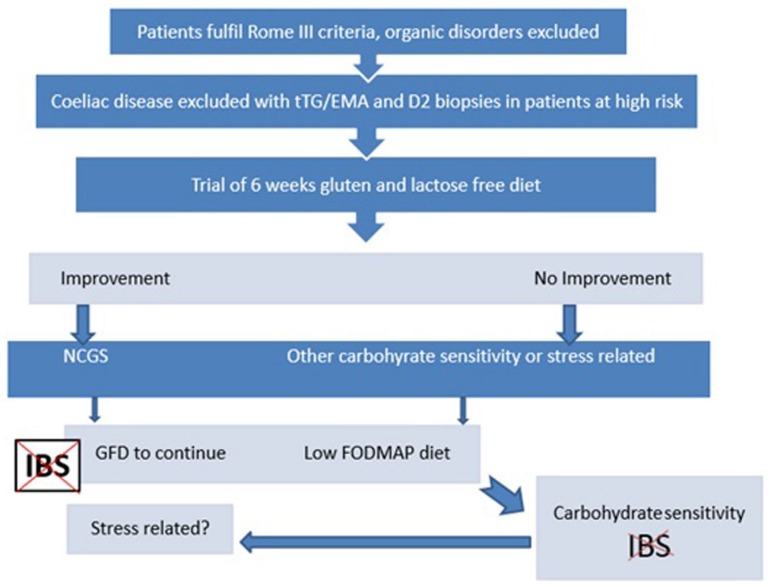
Suggesting algorithm.

In conclusion, many patients diagnosed as having IBS are clearly gluten-sensitive, and their symptoms could be adequately controlled with a gluten-free diet only. Identifying gluten sensitivity in this group of patients may need to be the first approach. Elimination of other carbohydrates might be considered in non-responders if six weeks of a gluten-free diet proves to be ineffective in controlling the patient’s symptoms, as according to Figure 3. Studies with a larger sample size would be needed to determine whether some biomarkers as predictors of response to treatment, may be useful in this subgroup of patients before a gluten-free diet.
